# NICU discharge preparation and transition planning: foreword

**DOI:** 10.1038/s41372-022-01311-x

**Published:** 2022-02-14

**Authors:** Heather Cohen Padratzik, Kristin Love

**Affiliations:** 1 Board of National Perinatal Association, St. Louis, MO USA; 2 National Perinatal Association, Lonedell, MO USA

## Abstract

Parents whose babies are admitted to the neonatal intensive care unit (NICU) need support. Whether their baby’s stay is brief or long, uncomplicated or complex, a NICU stay changes how they care for their infant and how they will parent once they are discharged. While we know a NICU stay is traumatic for most parents, the consequences of a family’s time spent in the NICU do not need to be negative ones. Supportive NICU teams can use the time a family is in the NICU to engage in a well-designed discharge preparation and transition planning program. These programs can have a lasting positive impact on both the infant’s health and the family’s wellbeing.

## Introduction

Having your baby admitted to the neonatal intensive care unit (NICU) to receive critical care is inherently traumatic. Few families anticipate their baby’s birth will become a medical emergency—and even fewer are familiar with the kind of care the NICU provides. Once there, parents begin to wonder if and when their baby will be able to come home. This is why we say that NICU discharge preparation and transition planning needs to begin on the first day of a family’s NICU stay. It is why we must remember that our goal is not just to send a baby home healthier than when they arrived, it is to send them home to a family that is empowered and prepared. If parents are going to become confident and competent caregivers for their infants they need guidance and support. The education they receive while in the NICU can not be limited to how to perform caregiving tasks. It has to expand to meet their need to become a parent to a medically-fragile child. It has to meet their social and emotional needs. It has to welcome them into a community of parents and providers. This is what smart, timely, coordinated care by an interdisciplinary NICU team can deliver.

## Heather

Being the parent of a baby just discharged home from the NICU is both exciting and terrifying. My story started back in 2012 when my son, Owen, was born at 28-weeks gestation. He was a tiny, fragile, two-pound preemie. After 81 very long days and nights, and many setbacks as well as met milestones, he was finally going home. I found out about his discharge only 2 days prior. Although I was overjoyed to find he was finally coming home with me, I was also incredibly overwhelmed at the thought of not being able to take with me all of the wonderful staff that had helped raise him and grow him into the strong baby he had become. Owen was to go home on a heart-apnea monitor to be sure we knew if he had any episodes. After only a 15-min explanation of how to operate the monitor and interpret its output, we were discharged to home with our new baby and this monitor a few hours later.

To say I was overjoyed was an understatement. However, I was also terrified that I might not know how to handle an episode on my own. I had experienced many episodes in the hospital where I was in his room when he stopped breathing and turned blue. Staff would come rushing into the room, surround him, and somehow, thankfully bring him out of each episode. This was an extremely frightening thing for a parent to witness. It occurred mostly when I would try to feed him. He would choke and turn blue. I had nightmares about these episodes while Owen was in the NICU and after. How would I ever be able to handle one on my own?

Each night during his stay, around 2:00 a.m., I would call Owen’s nurse to see how the night was progressing. I can still hear the hold music in my head today, as I waited anxiously for the nurse to answer the phone with a report. These anxious feelings took years to subside. The guilt I experienced as a preemie parent was overwhelming at times. Why couldn’t my body have kept my baby safe for a few more months so he didn’t have to experience all that he went through in the NICU? Did I eat something I shouldn’t have? Did I exercise too much? Each birthday celebration brought on a myriad of emotions. I was obviously ecstatic my little guy had grown up to be so strong and felt very fortunate he was not showing signs of long-term issues. However, each year I would also mourn the fact that I was not able to provide him a ‘normal’ term birth experience. I felt terrible that in his few years he had gone through so much.

Owen didn’t sleep much the first year, so I didn’t have time to manifest these feelings and deal with them until more than a year later. It became very clear from that point forward that it may have been beneficial if there had been an outlet for me to express these uncertain feelings and guilt. The support for my baby was amazing, with follow-up clinic visits and phone calls regarding his progress. However, the emotional toll I experienced was not addressed by any part of the process. To be a present and whole parent, I needed support to deal with these emotions and realize that they were a completely normal part of having had a premature baby.

These discharge guidelines include a social-emotional component that is extremely necessary and important for parents of preemies and medically-complex infants. Hospitals, physicians, and staff should recognize and address the potential emotional toll these transitions and experiences take on parents. Recognition of this and follow-up with caretakers as necessary will allow these individuals to be better parents in the long term.

## KRISTIN

My NICU journey began 21 years ago when my son, Travis was born at 30-weeks gestation and weighed a tiny three pounds. We soon learned how very fragile life is. Travis and I both experienced a lot of trauma during his 89-day stay; ventilator, open lung biopsy, resuscitated a half dozen times (three times before my very own eyes). With the support of my family, I was fortunate enough to be able to spend 8–10 h a day by his bedside. There, I learned how to care for my fragile baby with the amazing support and education of his medical team. Yet I will never forget the day my husband and I walked through the doors to find out Travis would be getting discharged in 48 h. We were overjoyed and terrified, all at the same time. Travis would be coming home on oxygen, an oxygen saturation monitor, and a heart-apnea monitor. We came in the next day to get our fifteen-minute crash course on how to use everything, during which I felt very overwhelmed. The staff continued to encourage me, “You have this. It is no different than what you have seen here. You just won’t see the numbers”. However, when we got home it was in fact very different. They were not there supporting me, reassuring me that I was doing everything right, and sticking their head in the door to confirm whether my baby’s coloring was ok or not. Everyone was gone. It was just Travis and me. It was now my job to keep my fragile baby alive.

Over the last 20 years of advocating for NICU families and babies, I have seen so many things change and improve. But we are still sending families home without appropriate support. I still get a sick feeling in my stomach when I hear a family share how terrified they were during and after discharge. I would ask myself, “Today, after all these years, why are families still feeling the way I did 21 years ago?”

## Conclusion

Our shared experiences illustrate that we could have benefitted even more from our families’ NICU journeys if things had been done differently during our discharge planning. We hope these guidelines will aid facilities in implementing a more robust discharge preparation and transition planning process. The experience of having a premature or medically fragile baby shapes and changes us forever. If those experiences are positive and empowering ones, we can use them to become better parents for our babies—no matter what their medical and developmental outcomes are.

